# Influenza Vaccination and Risk of Stroke in Women With Chronic Obstructive Pulmonary Disease: A Nationwide, Population-Based, Propensity-Matched Cohort Study

**DOI:** 10.3389/fmed.2022.811021

**Published:** 2022-05-19

**Authors:** Chun-Chao Chen, Cheng-Hsin Lin, Chun-Chih Chiu, Tsung Yeh Yang, Min-Huei Hsu, Yuan-Hung Wang, Meng-Huan Lei, Hsien Tang Yeh, Yu-Ann Fang, Wen-Rui Hao, Ju-Chi Liu

**Affiliations:** ^1^Division of Cardiology, Department of Internal Medicine, Shuang Ho Hospital, Taipei Medical University, New Taipei City, Taiwan; ^2^Taipei Heart Institute, Taipei Medical University, Taipei, Taiwan; ^3^Division of Cardiology, Department of Internal Medicine, School of Medicine, College of Medicine, Taipei Medical University, Taipei, Taiwan; ^4^Division of Cardiovascular Surgery, Department of Surgery, Shuang Ho Hospital, Taipei Medical University, New Taipei City, Taiwan; ^5^Division of Cardiovascular Surgery, Department of Surgery, School of Medicine, College of Medicine, Taipei Medical University, Taipei, Taiwan; ^6^Graduate Institute of Data Science, College of Management, Taipei Medical University, Taipei, Taiwan; ^7^Department of Neurosurgery, Wan-Fang Hospital, Taipei Medical University, Taipei, Taiwan; ^8^Department of Medical Research Executive Secretary, Center of Human Research Protection, Shuang Ho Hospital, Taipei Medical University, New Taipei City, Taiwan; ^9^Cardiovascular Center, Lo-Hsu Medical Foundation Lotung Poh-Ai Hospital, Luodong, Taiwan; ^10^Department of Surgery, Lotung Poh-Ai Hospital, Luodong, Taiwan; ^11^Graduate Institute of Clinical Medicine, College of Medicine, Taipei Medical University, Taipei, Taiwan

**Keywords:** women, COPD, influenza vaccination, ischemic stroke, hemorrhagic stroke

## Abstract

**Backgrounds:**

The risk of stroke is higher among patients with chronic obstructive pulmonary disease (COPD) than among the healthy population. Moreover, women generally have worse long-term stroke outcomes than men.

**Methods:**

The data of 6681 women with COPD (aged ≥ 65 years) registered in Taiwan’s National Health Insurance Research Database were retrospectively analyzed from January 1, 2001 to December 31, 2011. After 1:1 propensity score matching, the patients were divided into vaccinated and unvaccinated groups.

**Results:**

In total, 5102 women were enrolled. The vaccinated group had a significantly lower risk of total, hemorrhagic, and ischemic stroke than the unvaccinated group (adjusted hazard ratio [aHR]: 0.60, 95% confidence interval [CI]: 0.54–0.67; aHR: 0.59, 95% CI: 0.43–0.83; and aHR: 0.59, 95% CI: 0.52–0.68, respectively). A lower risk of stroke was observed among the women aged 65–74 and ≥75 years, and the association was dose-dependent in all types of stroke (aHR: 1.08, 95% CI: 0.92–1.26; aHR: 0.70, 95% CI: 0.60–0.82; and aHR: 0.32, 95% CI: 0.26–0.38 for those vaccinated 1, 2 to 3, and ≥4 times, respectively, during the follow-up period). Women with a CHA_2_DS_2_-VASc score (conditions and characteristics included congestive heart failure, hypertension, diabetes, stroke, vascular disease, age, and sex) of 2–3 and ≥4 had a significantly lower risk of ischemic stroke while receiving more vaccinations. A smaller significant lower risk of hemorrhagic stroke after more than 4 times of vaccination was noted in the women with a CHA_2_DS_2_-VASc score of ≥4. Both interrupted and non-interrupted vaccination was associated with lower risk of stroke occurrence.

**Conclusion:**

Influenza vaccination is associated with a lower risk of total, hemorrhagic, and ischemic stroke among women with COPD, and the association is dose-dependent. However, the findings may be limited by unmeasurable confounders. Further investigations on this subject are warranted.

## Introduction

Chronic obstructive pulmonary disease (COPD) is the third leading global cause of death (1). Although COPD is primarily considered a complex respiratory tract disease characterized by irreversible airway pathological changes, it is also strongly associated with cardiovascular disease (2, 3). Common comorbidities of cardiovascular disease include heart failure, ischemic heart disease, obesity, hypertension, hyperlipidemia, and diabetes (4). Furthermore, factors contributing to the development of cardiovascular disease include systemic inflammation, hypoxemia, oxidative stress, and arterial stiffness (5).

Cardiovascular disease risk and stroke prevalence and incidence are all significantly higher in patients with COPD than in the healthy population (6). Stroke is one of the leading causes of functional disability that results in reduced quality of life in patients with COPD and increases caregiver burden. Notably, long-term stroke outcomes are less favorable in women than in men (7).

Studies have identified associations between seasonal influenza infection and an increased risk of cardiovascular mortality (8, 9). A study on influenza vaccination and reduction in hospitalization for cardiac and stroke events among older adults observed that influenza vaccination had a cardioprotective effect on patients with COPD (10). Therefore, high-risk patients should receive annual influenza vaccination (10–12). Although influenza vaccination may reduce the risk of stroke in patients at high risk for thrombogenicity (13, 14), the effect of influenza vaccination on women with COPD remains unclear. Thus, the present study explored the potential cerebroprotective association between influenza vaccination and the risk of stroke among women with COPD.

## Materials and Methods

Taiwan’s National Health Insurance (NHI) program, launched in 1995, covers 98% of the population of Taiwan, which exceeds 23 million people. The NHI Research Database (NHIRD), which is maintained by the Health and Welfare Data Science Center, has been extensively analyzed and validated (13–16). In Taiwan, influenza vaccination is provided free of charge to adults aged older than 65 years with high-risk comorbidities (i.e., diabetes, chronic liver disease, cirrhosis, cardiovascular disease, and chronic pulmonary disease). All researchers using the NHIRD and its data subsets must sign a written agreement declaring that they have no intention of obtaining information that could potentially violate the privacy of patients or care providers.

### Study Cohort

The patients enrolled in the present study were women recorded as having COPD between January 1, 2001 and December 31, 2011, with all diagnoses corresponding to the codes of the *International Classification of Diseases, Ninth Revision, Clinical Modification* (*ICD-9-CM*). The patients must have been ≥65 years of age (*N* = 6681) and have had at least two COPD diagnoses as inpatients or outpatients (17). The positive predictive value of the *ICD-9-CM* codes in COPD diagnosis was previously validated (16). Vaccination status was identified by code V048 and/or the use of vaccine (confirmed by drug codes).

### Data Selection Process

**FIGURE 1:**
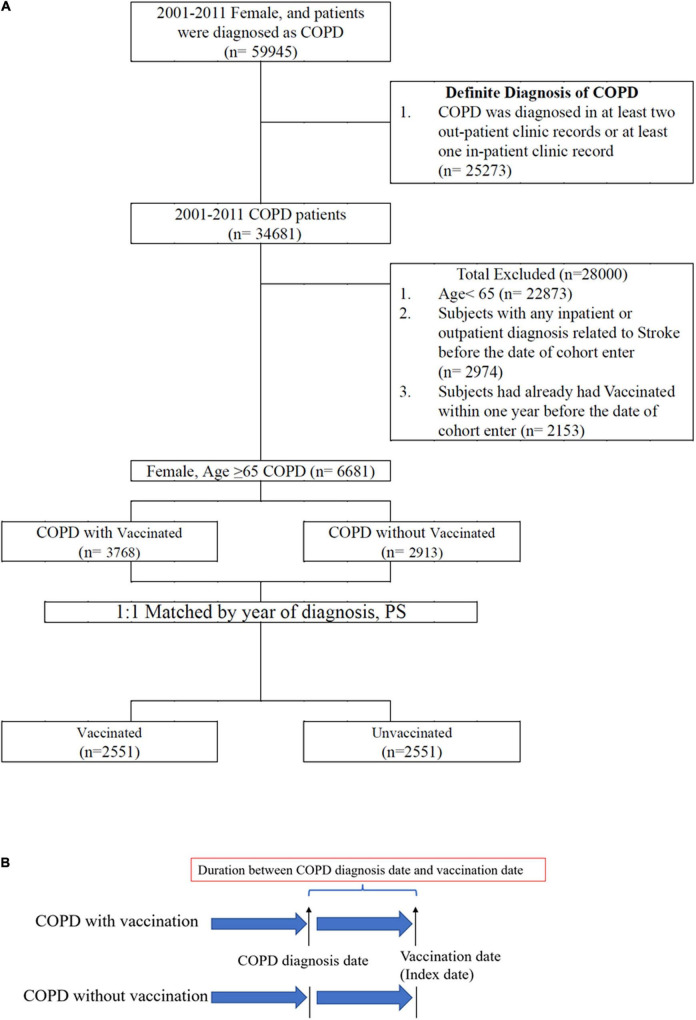
**(A)** Data selection process. **(B)** Definition of index date and duration between diagnosis date and vaccination date.

There were 3768 COPD patients who had vaccination and 2913 COPD patients who did not have vaccination. Propensity score matching (1:1) was performed and the patients were divided into vaccinated (*n* = 2551) and unvaccinated (*n* = 2551) groups (Figure 1A and Table 1).

**TABLE 1:** Pooled baseline characteristics, balanced before and after propensity score matching.

	Before matching					After matching				
	Unvaccinated (*n* = 2913)		Vaccinated (*n* = 3768)		Standardized difference	Unvaccinated (*n* = 2551)		Vaccinated (*n* = 2551)		Standardized difference
	*n*	%	*n*	%		*n*	%	*n*	%	
Propensity score (mean ± standard deviation)	0.53 ± 0.11		0.54 ± 0.10		0.095	0.54 ± 0.10		0.55 ± 0.10		0.010
**COPD-related inpatient visits**										
0	2222	76.28	2998	79.56	0.079	1972	77.30	1997	78.28	0.024
1	413	14.18	438	11.62	−0.076	315	12.35	313	12.27	−0.002
≥2	278	9.54	332	8.81	−0.025	264	10.35	241	9.45	−0.030
Age, years (mean ± standard deviation)	75.80 ± 7.60		73.59 ± 6.45		−0.282	74.91 ± 6.46		74.87 ± 6.46		0.006
65–74	1486	51.01	2422	64.28	0.271	1397	54.76	1433	56.17	0.028
≥75	1427	48.99	1346	35.72	−0.271	1154	45.24	1118	43.83	−0.028
**CHA2DS2-VASc score**										
2	369	12.67	585	15.53	0.083	351	13.76	320	12.54	−0.036
3	674	23.14	930	24.68	0.036	638	25.01	602	23.60	−0.033
≥4	1870	64.19	2253	59.79	−0.091	1562	61.23	1629	63.86	0.054
**Comorbidities**										
Asthma	1274	43.73	1953	51.83	0.163	1187	46.53	1289	50.53	0.080
HF	545	18.71	514	13.64	−0.138	393	15.41	394	15.44	0.001
AMI	62	2.13	61	1.62	−0.038	40	1.57	47	1.84	0.021
AF	419	14.38	506	13.43	−0.028	382	14.97	342	13.41	−0.045
Ischemic heart disease	1239	42.53	1383	36.70	−0.119	946	37.08	968	37.95	0.018
Angina	417	14.32	470	12.47	−0.054	290	11.37	329	12.90	0.047
Peripheral vascular disease	357	12.26	367	9.74	−0.081	250	9.80	257	10.07	0.009
Hypertension	2053	70.48	2477	65.74	−0.102	1645	64.48	1723	67.54	0.065
Diabetes	929	31.89	1106	29.35	−0.055	753	29.52	760	29.79	0.006
Depression	148	5.08	135	3.58	−0.074	103	4.04	102	4.00	−0.002
Renal failure	467	16.03	448	11.89	−0.120	358	14.03	335	13.13	−0.026
Chronic liver disease	752	25.82	863	22.90	−0.068	621	24.34	601	23.56	−0.018
Dementia	227	7.79	193	5.12	−0.109	135	5.29	169	6.62	0.056
**Urbanization level**										
Urban	1844	63.30	2236	59.34	−0.081	1593	62.45	1485	58.21	−0.087
Suburban	665	22.83	910	24.15	0.031	609	23.87	654	25.64	0.041
Rural	404	13.87	622	16.51	0.074	349	13.68	412	16.15	0.069
**Monthly income (NT$)**										
0	670	23.00	778	20.65	−0.057	650	25.48	561	21.99	−0.082
1–33 300	2060	70.72	2765	73.38	0.059	1795	70.36	1859	72.87	0.056
≥33 301	183	6.28	225	5.97	−0.013	106	4.16	131	5.14	0.047
**COPD medications**										
SABA	488	16.75	833	22.11	0.136	486	19.05	549	21.52	0.061
SABA + SAMA	375	12.87	523	13.88	0.030	321	12.58	350	13.72	0.034
LABA (ultra-LABA)	34	1.17	46	1.22	0.005	20	0.78	22	0.86	0.009
LAMA	50	1.72	92	2.44	0.050	47	1.84	57	2.23	0.028
ICS	200	6.87	367	9.74	0.104	188	7.37	237	9.29	0.070
ICS + LABA	264	9.06	467	12.39	0.108	247	9.68	308	12.07	0.077
**Total COPD medications**										
0	2042	70.10	2420	64.23	−0.125	1765	69.19	1646	64.52	−0.099
1	539	18.50	757	20.09	0.040	457	17.91	523	20.50	0.066
≥2	332	11.40	591	15.68	0.125	329	12.90	382	14.97	0.060
Medication use*										
Aspirin	962	33.02	1829	48.54	0.320	795	31.16	1014	39.75	0.180
Statin	658	22.59	1279	33.94	0.254	522	20.46	740	29.01	0.199
RAASi	1508	51.77	2478	65.76	0.287	1223	47.94	1498	58.72	0.217
Metformin	541	18.57	906	24.04	0.134	464	18.19	545	21.36	0.080

*COPD, chronic obstructive pulmonary disease; HF, heart failure; AMI, acute myocardial infarction; AF, atrial fibrillation; SABA, short-acting beta agonists; SAMA, short-acting muscarinic antagonists; LABA, long-acting beta agonists; ICS, inhaled corticosteroids; RAASi, renin–angiotensin–aldosterone system inhibitors.*

*The standardized difference is the difference in the mean or proportions divided by the standard error. Imbalance between groups was defined as an absolute value greater than 0.10 (corresponding to a small effect size).*

*In propensity score matching, adjustments were made for COPD-related inpatient visits, COPD medications, total COPD medications, age, CHA2DS2-VASc score, asthma, HF, AMI, AF, ischemic heart disease, angina, peripheral vascular disease, hypertension, diabetes, depression, renal failure, chronic liver disease, dementia, urbanization level, and monthly income.*

*These variables were not adjusted for in the propensity score matching.

### Index Date Definition

To avoid immortal time bias (18), the vaccination date of the patients in the vaccinated group was defined as the index or cohort entry date. In the matched pairs, the participants who did and did not receive vaccination were assigned the same index date (i.e., the vaccination date) for follow-up (Figure 1B).

### Definition of Interrupted and Non-interrupted Vaccination

Patients who received more than two vaccinations were divided into interrupted and non-interrupted groups. Interrupted vaccination was defined as receiving more than two vaccinations with any interruption during the follow-up period. Non-interrupted vaccination was defined as receiving more than two yearly vaccinations without any interruption during the follow-up period.

### Study Endpoint

The study endpoint was the initial diagnosis of hemorrhagic stroke (*ICD-9-CM* codes 430, 431, or 432), ischemic stroke (*ICD-9-CM* codes 433 or 434), or undefined stroke (*ICD-9-CM* code 436). All patients were followed until stroke diagnosis, withdrawal from NHI, loss to follow-up, death, or December 31, 2012. Except for those patients diagnosed with stroke, the other data were censored.

### Potential Confounders

The potential confounders of the cohort were based on sociodemographic characteristics (age, urbanization level, and monthly income), comorbidities [asthma, heart failure [HF], acute myocardial infarction [AMI], atrial fibrillation [AF], ischemic heart disease, angina, peripheral vascular disease, hypertension, diabetes, depression, renal failure, chronic liver disease, dementia, and CHA_2_DS_2_-VASc score (19)], COPD severity and treatment (COPD-related inpatient visits, COPD medications, total number of COPD medications) and medication use (aspirin, statins, renin–angiotensin–aldosterone system inhibitors [RAASi], and metformin). These confounders have been implicated as possible risk factors in stroke (19–21).

### Matching Factors

Propensity score matching, which involves assigning levels of 0 and 1 to a treatment variable, given a set of known variables, is used to adjust for potential selection bias, confounding, and differences between treatment groups in observational studies (22). In the present study, the propensity score of each vaccinated patient was estimated by logistic regression with the following 25 potential confounders associated with vaccine introduction: sociodemographic characteristics (age, urbanization level, and monthly income), comorbidities [asthma, HF, AMI, AF, ischemic heart disease, angina, peripheral vascular disease, hypertension, diabetes, depression, renal failure, chronic liver disease, dementia, and CHA_2_DS_2_-VASc score (19)], and COPD severity and treatment (COPD-related inpatient visits, COPD medications, and total number of COPD medications). The vaccinated and unvaccinated patients were then matched using propensity scores and a 1:1 nearest neighbor algorithm. As previously suggested (23), the caliper width was set as 0.03 of the pooled standard deviation of the logit of the propensity scores. Finally, the patients were divided into vaccinated (*n* = 2551) and unvaccinated (*n* = 2551) groups.

### Statistical Analysis

In the present study, categorical data are expressed as numbers and percentages, whereas quantitative data are presented as the means ± standard deviations. The balance of characteristics was assessed by estimating the standardized differences (StDiffs) between the vaccinated and unvaccinated groups. Empirically, an absolute value of StDiffs that exceeds 0.1 (10%) represents a meaningful imbalance in a given variable between two groups. A Cox proportional hazards model was used to calculate the hazard ratios (HRs) to determine the differences in the risk of stroke between the groups. The adjusted HRs (aHRs) were HRs that were adjusted according to the confounder propensity score. In addition to the previously mentioned confounders, the duration between the COPD diagnosis date and index date was also included in the Cox proportional hazard model for aHR analysis. Fine and Gray’s (F&G) survival and regression analyses were used to determine the risk of stroke while competing with death. In this way the F&G method models the subdistribution hazards. The effect estimated using the F&G model shows the current and real differences between the treatment groups in terms of subdistribution hazards ratios. The assumption of proportionality of hazards is still a requirement, but of course it refers to the subdistribution hazards. The F&G model can accommodate time dependent coefficients to model the non-proportionality of hazards. This model can be applied to both the event of interest (stroke) or the competing risk (death). Sensitivity analysis can improve the understanding of the effects of drugs and biologics in epidemiologic database studies (24). Thus, in the present sensitivity analysis, the patients were stratified to estimate the association of age, CHA_2_DS_2_-VASc score, COPD-related inpatient visits, asthma, HF, AF, ischemic heart disease, angina, peripheral vascular disease, hypertension, diabetes, renal failure, and chronic liver disease with the incidence of stroke in different models. The cumulative incidence of stroke in the vaccinated and unvaccinated patients with COPD was calculated using the cumulative incidence function. All analyses were performed using SAS, version 9.4 (SAS Institute Inc., Cary, NC, United States). A two-tailed *P*-value of <0.05 was considered significant.

## Results

The baseline characteristics of both groups of patients after propensity score matching are presented in Table 1. As mentioned, 6681 patients were enrolled, and after 1:1 propensity score matching, we divided the patients into vaccinated and unvaccinated groups (each group, *n* = 2551).

### The Association Between Influenza Vaccination and Stroke Occurrence in Different Age Groups and Patients With Varying CHA_2_DS_2_-VASc Scores

**FIGURE 2:**
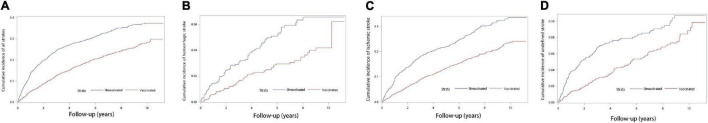
Cumulative incidence rates of different types of strokes estimated by the cumulative incidence function competing risk analysis between patients with and without vaccination. **(A)** Overall stroke events (χ^2^ = 79.308; df = 1; *P* < 0.001). **(B)** Hemorrhagic stroke events (χ^2^ = 13.466; df = 1; *P* < 0.001). **(C)** Ischemic stroke events (χ^2^ = 81.272; df = 1; *P* < 0.001). **(D)** Undefined stroke events (χ^2^ = 18.660; df = 1; *P* < 0.001).

Table 2 shows the association between influenza vaccination and the risk of stroke in the present cohort, stratified by age and CHA_2_DS_2_-VASc score. Ischemic stroke was higher in incidence than hemorrhagic stroke during the follow-up period. Overall, the occurrence of all strokes, hemorrhagic, ischemic, and undefined were significantly lower in the vaccinated group (aHR: 0.60, 95% confidence interval [CI]: 0.54–0.67; aHR: 0.59, 95% CI: 0.43–0.83; aHR: 0.59, 95% CI: 0.52–0.68; and aHR: 0.64, 95% CI: 0.50–0.81, respectively) than in the unvaccinated group (Figures 2A–D). In the Fine and Gray competing risk model, the risk of all strokes remained significantly lower in the vaccinated group while competing with death (Table 2). Influenza vaccination was also associated with a lower risk of hemorrhagic, ischemic, and undefined stroke in patients aged 65 to 74 years. Vaccinated patients aged ≥75 years had a lower risk of all strokes and ischemic stroke than their unvaccinated counterparts.

**TABLE 2:** Risk of stroke in the unvaccinated and vaccinated groups.

Study Cohort (*N* = 5102)		Unvaccinated (total follow-up duration: 10028.9 person-years)					Vaccinated (total follow-up duration: 12858.0 person-years)			HR (95% CI)	aHR^†^ (95% CI)	Subdistribution HR^‡^ (95% CI)	
	Death Before Event	Number of Events	Incidence Rate (per 10^5^ person-years) (95% CI)			Death Before Event	Number of Events	Incidence Rate (per 10^5^ person-years) (95% CI)					
**Entire cohort**													
Stroke	595	770	7677.8	(7135.5,	8220.1)	525	537	4176.4	(3823.1,	4529.6)	0.57 (0.51, 0.64)***	0.60 (0.54, 0.67)***	0.64 (0.58, 0.72)***
Hemorrhagic stroke	909	90	897.4	(712.0,	1082.8)	705	61	474.4	(355.4,	593.5)	0.55 (0.45, 0.76)***	0.59 (0.43, 0.83)**	0.67 (0.48, 0.92)*
Ischemic stroke	716	516	5145.1	(4701.2,	5589.1)	606	357	2776.5	(2488.5,	3064.5)	0.56 (0.49, 0.64)***	0.59 (0.52, 0.68)***	0.65 (0.57, 0.74)***
Undefined stroke	874	164	1635.3	(1385.0,	1885.6)	686	119	925.5	(759.2,	1091.8)	0.60 (0.47, 0.76)***	0.64 (0.50, 0.81)***	0.71 (0.56, 0.90)**
**Age, 65–74 years** ^a^													
Stroke	237	395	6468.6	(5830.7,	7106.5)	178	283	3569.2	(3153.4,	3985.1)	0.57 (0.49, 0.66)***	0.60 (0.50, 0.70)***	0.64 (0.55, 0.74)***
Hemorrhagic stroke	340	50	818.8	(591.8,	1045.8)	241	31	391.0	(253.3,	528.6)	0.50 (0.32, 0.78)**	0.54 (0.34, 0.84)**	0.59 (0.38, 0.93)*
Ischemic stroke	287	252	4126.8	(3617.3,	4636.3)	208	193	2434.1	(2090.7,	2777.6)	0.61 (0.50, 0.73)***	0.63 (0.52, 0.76)***	0.68 (0.57, 0.83)***
Undefined stroke	320	93	1523.0	(1213.5,	1832.5)	233	59	744.1	(554.2,	934.0)	0.51 (0.37, 0.71)***	0.55 (0.39, 0.76)***	0.60 (0.43, 0.82)**
**Age, ≥ 75 years** ^b^													
Stroke	358	375	9560.2	(8592.6,	10527.9)	347	254	5153.1	(4519.3,	5786.8)	0.57 (0.48, 0.67)***	0.62 (0.52, 0.72)***	0.65 (0.56, 0.77)***
Hemorrhagic stroke	569	40	1019.8	(703.7,	1335.8)	464	30	608.6	(390.8,	826.4)	0.62 (0.38, 0.99)*	0.65 (0.40, 1.06)	0.75 (0.47, 1.21)
Ischemic stroke	429	264	6730.4	(5918.5,	7542.3)	398	164	3327.2	(2818.0,	3836.4)	0.52 (0.43, 0.63)***	0.57 (0.47, 0.69)***	0.62 (0.51, 0.75)***
Undefined stroke	554	71	1810.1	(1389.0,	2231.1)	453	60	1217.3	(909.3,	1525.3)	0.71 (0.51, 1.01)	0.76 (0.53, 1.07)	0.86 (0.61, 1.20)
**CHA2DS2-VASc = 2–3^c^**													
Stroke	224	262	5560.2	(4886.9,	6233.4)	154	159	2861.4	(2416.6,	3306.2)	0.53 (0.43, 0.64)***	0.56 (0.46, 0.68)***	0.61 (0.50, 0.74)***
Hemorrhagic stroke	287	40	848.9	(585.8,	1111.9)	187	21	377.9	(216.3,	539.6)	0.45 (0.27, 0.77)**	0.51 (0.30, 0.87)*	0.57 (0.34, 0.96)*
Ischemic stroke	255	168	3565.3	(3026.2,	4104.4)	168	106	1907.6	(1544.5,	2270.8)	0.55 (0.43, 0.70)***	0.58 (0.46, 0.74)***	0.64 (0.51, 0.82)***
Undefined stroke	294	54	1146.0	(840.3,	1451.6)	185	32	575.9	(376.3,	775.4)	0.52 (0.33, 0.80)**	0.52 (0.34, 0.82)**	0.58 (0.37, 0.89)*
**CHA2DS2-VASc ≥ 4^d^**													
Stroke	371	508	9554.6	(8723.7,	10385.5)	371	378	5177.2	(4655.2,	5699.1)	0.57 (0.50, 0.65)***	0.61 (0.54, 0.70)***	0.66 (0.58, 0.75)***
Hemorrhagic stroke	622	50	940.4	(679.7,	1201.1)	518	40	547.8	(378.1,	717.6)	0.61 (0.40, 0.93)*	0.65 (0.43, 0.99)*	0.74 (0.49, 1.13)
Ischemic stroke	461	348	6545.3	(5857.6,	7233.0)	438	251	3437.7	(3012.4,	3863.0)	0.55 (0.47, 0.65)***	0.59 (0.50, 0.69)***	0.65 (0.55, 0.76)***
Undefined stroke	580	110	2068.9	(1682.3,	2455.5)	501	87	1191.6	(941.2,	1442.0)	0.61 (0.46, 0.82)***	0.68 (0.51, 0.90)**	0.77 (0.58, 1.02)

*^a^Total follow-up duration: 6106.4 and 7928.9 for the unvaccinated and vaccinated groups, respectively.*

*^b^Total follow-up duration: 3922.5 and 4929.1 person-years for the unvaccinated and vaccinated groups, respectively.*

*^c^Total follow-up duration: 4712.1 and 5556.7 person-years for the unvaccinated and vaccinated groups, respectively.*

*^d^Total follow-up duration: 5316.8 and 7301.3 person-years for the unvaccinated and vaccinated groups, respectively. CI, confidence interval; HR, hazard ratio; aHR, adjusted HR; HF, heart failure; AMI, acute myocardial infarction; AF, atrial fibrillation; RAASi, renin–angiotensin–aldosterone system inhibitors.*

*^†^The main model was adjusted for COPD-related inpatient visits, COPD medications, total COPD medications, age, CHA2DS2-VASc score, asthma, HF, AMI, AF, ischemic heart disease, angina, peripheral vascular disease, hypertension, diabetes, depression, renal failure, chronic liver disease, dementia, level of urbanization, monthly income, and the use of aspirin, statins, RAASi, and metformin, duration between the COPD diagnosis date and index date.*

*^‡^A subdistribution hazard model was used as a sensitivity analysis to account for death as a competing risk.*

*P < 0.05; **P < 0.01; ***P < 0.001.

The occurrence of hemorrhagic, ischemic, and undefined stroke was considerably lower in women with COPD and a CHA_2_DS_2_-VASc score of 2–3 after they were vaccinated. Compared with that of women with a CHA_2_DS_2_-VASc score of 2–3, the risk of ischemic stroke was significantly lower in vaccinated women with COPD who had higher CHA_2_DS_2_-VASc scores.

### Association Between the Total Number of Influenza Vaccinations and Stroke Risk

**FIGURE 3:**
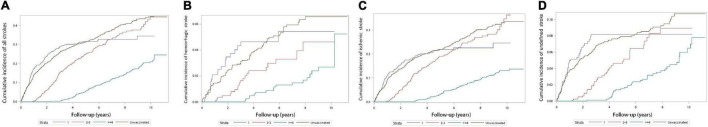
Cumulative incidence rates of different types of strokes estimated by the cumulative incidence function competing risk analysis between patients total number of vaccinations **(A)** Overall stroke events (χ^2^ = 217.451; df = 3; *P* < 0.001). **(B)** Hemorrhagic stroke events (χ^2^ = 27.725; df = 3; *P* < 0.001). **(C)** Ischemic stroke events (χ^2^ = 153.256; df = 3; *P* < 0.001). **(D)** Undefined stroke events (χ^2^ = 39.481; df = 3; *P* < 0.001).

In the main model, a higher number of influenza vaccinations over the follow-up period was associated with a lower risk of stroke occurrence in women with COPD overall (aHR: 1.08, 95% CI: 0.92–1.26; aHR: 0.70, 95% CI: 0.60–0.82; and aHR: 0.32, 95% CI: 0.26–0.38 for patients vaccinated 1, 2 to 3, and ≥4 times, respectively; Table 3 and Figure 3A). Patients with a history of asthma, HF, AF, ischemic heart disease, angina, hypertension, diabetes, renal failure, and chronic liver disease had a reduced risk of developing all types of stroke after they received more vaccinations. Notably, a considerably lower risk of all types of stroke was detected in patients without such history after they received more vaccinations. In patients with a CHA_2_DS_2_-VASc score 2–3, significantly lower risk of stroke after receiving more than four times of vaccination was observed. In patients with a CHA_2_DS_2_-VASc score ≥ 4, a substantially lower risk of all types of stroke was observed after more than two vaccinations, and further reductions in risk were noted in subsequent vaccinations, with a significant trend (Table 3). The patients with no COPD-related inpatient visits had a substantially lower risk of all types of strokes after receiving more than two vaccinations. The same results were noted in the patients with one COPD-related inpatient visit. Among patients with two or more COPD-related inpatient visits, the risk of all types of strokes only decreased after they received four or more vaccinations (Table 3). In patients who did not have influenza during the follow-up period, a substantially lower risk of all types of stroke was observed after vaccination. In patients who had influenza infection during the follow-up period, a significant trend of lower risk of all types of stroke was observed after vaccination (Table 3).

**TABLE 3:** Sensitivity analysis of the adjusted hazard ratios of vaccination in stroke risk reduction.

	Unvaccinated	Vaccinated			*P* for Trend
		1	2–3	≥4	
					
	aHR (95% CI)	aHR (95% CI)	aHR (95% CI)	aHR (95% CI)	
**Unadjusted**	1.00	1.06 (0.90, 1.24)	0.68 (0.59, 0.79)***	0.29 (0.24, 0.35)***	<0.001
Main model†	1.00	1.08 (0.92, 1.26)	0.70 (0.60, 0.82)***	0.32 (0.26, 0.38)***	<0.001
Competing Risk model^‡^	1.00	0.94 (0.80, 1.12)	0.73 (0.63, 0.85)***	0.39 (0.33, 0.46)***	<0.001
**Subgroup effects**					
Age, years					
65–74	1.00	1.04 (0.81, 1.33)	0.81 (0.66, 1.00)*	0.37 (0.30, 0.47)***	<0.001
≥75	1.00	0.87 (0.69, 1.09)	0.66 (0.53, 0.82)***	0.42 (0.32, 0.55)***	<0.001
**COPD-related inpatient visits**					
0	1.00	0.88 (0.72, 1.08)	0.77 (0.65, 0.92)**	0.37 (0.30, 0.45)***	<0.001
1	1.00	0.96 (0.64, 1.46)	0.55 (0.36, 0.84)**	0.38 (0.22, 0.65)***	<0.001
≥2	1.00	1.08 (0.72, 1.62)	0.66 (0.43, 1.02)	0.55 (0.34, 0.88)*	0.005
**CHA2DS2-VASc score**					
2–3	1.00	0.78 (0.54, 1.12)	0.84 (0.64, 1.09)	0.40 (0.30, 0.53)***	<0.001
≥4	1.00	0.93 (0.77, 1.13)	0.67 (0.56, 0.80)***	0.40 (0.32, 0.50)***	<0.001
**Asthma**					
No	1.00	0.83 (0.65, 1.06)	0.74 (0.60, 0.91)**	0.32 (0.25, 0.42)***	<0.001
Yes	1.00	1.05 (0.83, 1.32)	0.72 (0.59, 0.89)**	0.44 (0.36, 0.56)***	<0.001
**HF**					
No	1.00	0.92 (0.77, 1.12)	0.76 (0.65, 0.89)***	0.40 (0.33, 0.48)***	<0.001
Yes	1.00	1.00 (0.69, 1.43)	0.62 (0.42, 0.91)*	0.33 (0.20, 0.55)***	<0.001
**AF**					
No	1.00	0.97 (0.81, 1.17)	0.76 (0.65, 0.90)**	0.38 (0.31, 0.46)***	<0.001
Yes	1.00	0.82 (0.56, 1.20)	0.66 (0.47, 0.92)*	0.50 (0.35, 0.71)***	<0.001
**Ischemic heart disease**					
No	1.00	0.93 (0.75, 1.16)	0.75 (0.62, 0.91)**	0.38 (0.31, 0.47)***	<0.001
Yes	1.00	0.92 (0.71, 1.19)	0.68 (0.54, 0.86)**	0.40 (0.29, 0.53)***	<0.001
**Angina**					
No	1.00	0.89 (0.74, 1.06)	0.75 (0.64, 0.87)***	0.37 (0.31, 0.45)***	<0.001
Yes	1.00	1.49 (0.96, 2.30)	0.65 (0.41, 1.04)	0.53 (0.32, 0.88)*	0.005
**Peripheral vascular disease**					
No	1.00	0.96 (0.80, 1.15)	0.72 (0.62, 0.84)***	0.37 (0.31, 0.44)***	<0.001
Yes	1.00	0.81 (0.48, 1.38)	0.84 (0.53, 1.34)	0.68 (0.39, 1.18)	0.153
**Hypertension**					
No	1.00	0.86 (0.61, 1.21)	0.77 (0.58, 1.01)	0.44 (0.33, 0.58)***	<0.001
Yes	1.00	0.92 (0.76, 1.12)	0.70 (0.59, 0.84)***	0.38 (0.31, 0.47)***	<0.001
**Diabetes**					
No	1.00	0.85 (0.69, 1.06)	0.70 (0.59, 0.84)***	0.39 (0.32, 0.48)***	<0.001
Yes	1.00	1.10 (0.84, 1.45)	0.78 (0.60, 1.02)	0.39 (0.28, 0.53)***	<0.001
**Renal failure**					
No	1.00	0.92 (0.77, 1.11)	0.72 (0.61, 0.84)***	0.38 (0.32, 0.46)***	<0.001
Yes	1.00	1.05 (0.70, 1.57)	0.78 (0.54, 1.14)	0.42 (0.26, 0.68)***	<0.001
**Chronic liver disease**					
No	1.00	0.89 (0.74, 1.08)	0.71 (0.60, 0.84)***	0.40 (0.33, 0.49)***	<0.001
Yes	1.00	1.12 (0.80, 1.57)	0.82 (0.59, 1.13)***	0.32 (0.22, 0.48)***	<0.001
**Influenza infection**					
No	1.00	0.91 (0.75, 1.10)	0.72 (0.61, 0.86)***	0.42 (0.35, 0.51)***	<0.001
Yes	1.00	1.10 (0.76, 1.61)	0.69 (0.51, 0.92)*	0.27 (0.19, 0.38)***	<0.001

*P < 0.05; **P < 0.01; ***P < 0.001. COPD, chronic obstructive pulmonary disease; CI, confidence interval; aHR, adjusted hazard ratio; HF, heart failure; AMI, acute myocardial infarction; AF, atrial fibrillation; RAASi, renin–angiotensin–aldosterone system inhibitors.

*^†^The main model was adjusted for COPD-related inpatient visits, COPD medications, total COPD medications, age, CHA2DS2-VASc score, asthma, HF, AMI, AF, ischemic heart disease, angina, peripheral vascular disease, hypertension, diabetes, depression, renal failure, chronic liver disease, dementia, urbanization level, monthly income, and the use of aspirin, statins, RAASi, and metformin, duration between the COPD diagnosis date and index date.*

*^‡^A subdistribution hazard model was used as a sensitivity analysis to account for death as a competing risk.*

Supplementary Tables 1–3 present the sensitivity analysis results of the total number of vaccinations in patients with hemorrhagic, ischemic, and undefined stroke, respectively. The main model analysis revealed that patients who received 2 to 3 and ≥ 4 vaccinations had a lower risk of developing these types of strokes (Supplementary Tables 1–3 and Figures 3B–D). Among patients with no COPD-related inpatient visits, the risk of hemorrhagic stroke decreased after they received more than 4 vaccinations. Following multiple vaccinations, patients without HF, ischemic heart disease, angina, peripheral vascular disease, diabetes, renal failure, or chronic liver disease were less likely to develop hemorrhagic stroke. After receiving more than four vaccinations, patients with hypertension had a reduced risk of hemorrhagic stroke. All the patients had a reduced risk or developed ischemic stroke after receiving a higher number of vaccinations, regardless of the presence of those comorbidities, except patients with angina. Among women with a CHA_2_DS_2_-VASc score ≥ 4, the risk of hemorrhagic stroke decreased following more than 4 times of vaccinations, but the risk of ischemic stroke decreased after receiving more than two times of vaccinations. Among patients with a CHA_2_DS_2_-VASc score 2–3, the risk of hemorrhagic stroke did not decrease despite receiving more than four times of vaccination. A lower risk of ischemic stroke and undefined stroke occurrence was observed in patients with a CHA_2_DS_2_-VASc score of 2 to 3, or ≥4 after they received 4 or more vaccinations.

Analysis revealed that both interrupted and non-interrupted influenza vaccinations (Supplementary Tables 4–6) were associated with a lower risk of all strokes. In the sensitivity analysis, the risk of stroke was significantly lower after receiving more vaccination regardless of influenza infection (Table 3 and Supplementary Tables 1–3). In mediator analysis of present study, there were no significant decreasing influenza infection after influenza vaccination (β = −0.129) nor significant occurrence of stroke after influenza infection (β = 0.127). However, there was a significant stroke risk reduction after influenza vaccination (β = −0.567^***^, *p* < 0.001). The results of mediator analysis suggested that influenza infection was not a mediator effect in the present study (Supplementary Figure).

## Discussion

### Main Findings

The main findings of the present nationwide population-based propensity-matched cohort study are as follows: (1) Overall, women with COPD had a potentially lower risk of developing any of the types of stroke after receiving influenza vaccination. A similar association between a lower risk of stroke and influenza vaccination was found in elderly (≥65 years-old) and late elderly (≥75 years-old) women (2). The association between influenza vaccination and a lower risk of stroke in women with COPD appeared to be dose-dependent and consistent across all types of stroke (3). Regardless of the presence of comorbidities, women with COPD had a significantly lower risk of ischemic stroke after receiving more than four influenza vaccinations. However, only women with COPD without comorbidities except hypertension had a lower risk of hemorrhagic stroke after receiving more than four influenza vaccinations during the follow-up period (4). Among women with COPD and a CHA_2_DS_2_-VASc score of 2 to 3, receiving more than four vaccinations was associated with a significantly lower risk of stroke. Women with COPD and CHA_2_DS_2_-VASc scores of ≥4 all had a significantly lower risk of stroke after receiving more than two influenza vaccinations.

### Association Between Influenza Vaccination and Stroke in Women With Chronic Obstructive Pulmonary Disease

Influenza vaccination is strongly associated with a reduced risk of ischemic and hemorrhagic stroke (10, 13, 14, 25). In a recent nationwide observational study from Spain (26), a significantly higher risk of in-hospital mortality and mechanical ventilation usage was noted among women with COPD who were admitted because of ischemic stroke. Therefore, decreasing the risk of stroke in women with COPD is important. However, few studies have addressed the benefits of influenza vaccination in women with COPD.

The literature indicates the existence of sex differences in stroke. In general, women who have had a stroke are older and have more disabilities (7). Experimental studies have demonstrated the potential cardioprotective effect of estrogen (27). Therefore, postmenopausal women have a higher risk of stroke than premenopausal women (28). In Taiwan, influenza vaccination is provided free of charge to patients aged ≥65 years with high-risk comorbidities, which explains why the majority of the patients in the present study were postmenopausal. After adjustment for comorbidities, medications, urbanization level, monthly income, COPD-related inpatient visits, duration between COPD diagnosis date and vaccination date and age, influenza vaccination remained associated with a lower risk of all types of stroke. Furthermore, the risk of stroke remained significantly lower among older adults aged ≥75 years after receiving vaccination. In addition, a potential protective association was observed in both patients who did and did not have influenza.

Possible explanations are as follows: First, viral infection may trigger the thrombogenic process and induce coagulopathy (29). Influenza vaccination prevents postviral infection thrombus and hemorrhage. In addition, influenza infection may contribute to carotid atherosclerotic plaque inflammation and the eventual development of acute vascular events (30). Furthermore, lowering the risk of influenza infection prevents relevant consequences such as elevated sympathetic tone, hypoxemia, and endothelial dysfunction (31). Second, in a study on apoE knockout mice, influenza vaccination promoted the formation of smaller and more stable atherosclerotic plaques through an immunoresponse (32). Moreover, decreasing levels of interferon gamma, interleukin 2, and tumor necrosis factor alpha, as well as the activation of the bradykinin 2 receptor signaling pathway, constitute potential mechanisms underlying the stabilization of atherosclerotic plaques by influenza vaccination (32, 33).

As mentioned, the association between vaccination and a lower risk of stroke observed in the present study appeared to be dose-dependent and did not differ depending on whether multiple vaccinations were administered consecutively or were interrupted. A higher number of vaccinations was significantly associated with a reduction in all types of stroke, a result in line with that of another study (34). A systematic review and meta-analysis indicated that the protective effects of the influenza vaccine against different types of viruses may be less notable following repeated vaccinations (35). However, annual vaccination is still recommended for the prevention and control of influenza infections (36). The fact that vaccine effectiveness in the United States during the 2017–2018 influenza season was only 38% (37) suggests that receiving vaccination only once may not be sufficient to prevent contracting the virus. With more than one vaccination, the risk of influenza infection may also decrease, as may the risk of further cardiovascular complications, including stroke.

We further investigated the effect of the CHA_2_DS_2_-VASc score in women with COPD. Higher CHA_2_DS_2_-VASc scores were associated with a higher risk of ischemic stroke in patients with COPD, even those without AF (19). Notably, although the sensitivity analysis yielded significant results for both ischemic and hemorrhagic stroke, vaccination had a stronger risk reduction effect on ischemic stroke in the patients with CHA_2_DS_2_-VASc scores of ≥2. Because most hemorrhagic strokes are caused by ruptured cerebral vessels resulting from uncontrolled hypertension and abnormal cerebrovascular structure, the protective effect of influenza vaccination with plaque stabilization (32, 33) may be diminished in patients with these conditions. However, among female COPD patients with hypertension, the potentially risk reduction of hemorrhagic stroke was observed in the present study. Prospective studies are warranted to confirm these suppositions.

In addition, a significantly lower risk of stroke was observed in the patients with more inpatient visits because of the acute deterioration of their condition after they received four vaccinations, which is a greater number of vaccinations than that required for a protective effect against stroke in the patients with fewer inpatient visits. Thus, acute exacerbation in patients with COPD must be prevented to reduce complications.

In Taiwan, annual influenza vaccination constitutes a preventive strategy against an influenza epidemic. Previous nationwide studies conducted in Taiwan have indicated that influenza vaccination has benefits on cardiovascular and neurological outcomes (10, 14, 38). Overall, the present findings serve as a reference for policy-making with regard to annual influenza vaccination, especially in individuals with high-risk comorbidities.

### Limitations

This study has several limitations. First, its retrospective nature limits the generalizability of the findings; prospective randomized controlled trials are required to confirm the present results. Second, the NHIRD does not contain definite information on the severity classification of COPD as indicated by physical activity, smoking status, alcohol intake, body mass index, spirometry test results, and other laboratory data. We performed propensity matching to minimize the impact of this limitation (22). Third, healthy user bias may have been present (39). Therefore, we adjusted for sociodemographic characteristics, such as urbanization and monthly income, as potential confounders. In addition, because influenza vaccination in Taiwan was provided free of charge to the members of our cohort, vaccination refusal caused by the inability to pay for the procedure seems unlikely; as mentioned, the influenza vaccine coverage rate among older adults in Taiwan has reached 49% in recent years (40). Fourth, the data of the present study were collected until the end of 2011, and future studies enrolled more recent data to validate the results of the present study. Moreover, although we controlled for and minimized the bias by propensity score matching and sensitivity analysis, the bias from the residual unmeasured confounders and healthy user bias could still present. Fifth, the patients enrolled in the present study were based on the ICD-9 diagnosis. Although the positive predictive rate and accuracy had been validated before (16, 17), the potential bias from patient selection process remained exist, which could influence the result of the present study.

In conclusion, influenza vaccination was associated with a considerably lower risk of ischemic, hemorrhagic, and undefined stroke in women with COPD, and the association appeared to be dose-dependent. Among women with a CHA_2_DS_2_-VASc score of ≥2, the association between vaccination and hemorrhagic stroke risk was not as substantial as ischemic stroke. Further investigations are required to determine the potential mechanism of influenza vaccination against stroke in this patient population.

## Data Availability Statement

The data supporting the findings of this research were sourced from NHIRD in Taiwan. Owing to the legal restrictions imposed by the Government of Taiwan related to the Personal Information Protection Act, the database cannot be made publicly available.

## Ethics Statement

The present study protocol was approved by the NHIRD research committee and the Taipei Medical University Joint Institutional Review Board (TMU-JIRB No. N201804043).

## Author Contributions

W-RH and J-CL are the guarantors of the content of the manuscript and including the data and analysis. C-CChen conceived and designed the study and drafted the manuscript. Y-AF was responsible for data collection. Y-AF, C-HL, W-RH, C-CChiu, T-YY, M-HH, M-HL, H-TY, and Y-HW analyzed and interpreted the data. All authors reviewed the manuscript and approved its submission.

## Conflict of Interest

The authors declare that the research was conducted in the absence of any commercial or financial relationships that could be construed as a potential conflict of interest.

## Publisher’s Note

All claims expressed in this article are solely those of the authors and do not necessarily represent those of their affiliated organizations, or those of the publisher, the editors and the reviewers. Any product that may be evaluated in this article, or claim that may be made by its manufacturer, is not guaranteed or endorsed by the publisher.
