# Supplementary material for: Influenza Vaccination and Risk of Stroke in Women With Chronic Obstructive Pulmonary Disease: A Nationwide, Population-Based, Propensity-Matched Cohort Study
Source: Front Med (Lausanne). 2022 May 19;9:811021. doi: 10.3389/fmed.2022.811021 (PMC9160371; doi:10.3389/fmed.2022.811021)
Supplement: Supplementary file 1 [file Data_Sheet_1.docx]

**Table 1.** **Sensitivity Analysis of the Adjusted Hazard Ratios of Vaccination in Hemorrhagic Stroke Risk Reduction**

|  | | Unvaccinated | | Vaccinated | | | | *P* for Trend |  |
| --- | --- | --- | --- | --- | --- | --- | --- | --- | --- |
|  |  |  |  | 1 | | 2–3 | **≥**4 |  |  |
|  |  | aHR  (95% CI) | | aHR  (95% CI) | | aHR  (95% CI) | aHR  (95% CI) |  |  |
| **Unadjusted** | | 1.00 | | 1.15 (0.73, 1.81) | | 0.55 (0.34, 0.90)* | 0.31(0.18, 0.52)*** | < .001 |  |
| Main model † | | 1.00 | | 1.18 (0.75, 1.85) | | 0.58 (0.36, 0.94)* | 0.35(0.21, 0.59)*** | < .001 |  |
| Competing Risk model‡ | | 1.00 | | 1.03 (0.66, 1.62) | | 0.64 (0.39, 1.03) | 0.46(0.28, 0.75)** | < .001 |  |
| **Subgroup effects** | |  | |  | |  |  |  |  |
| Age, years | |  | |  | |  |  |  |  |
| 65–74 | | 1.00 | | 0.83 (0.41, 1.70) | | 0.66 (0.34, 1.27) | 0.44(0.24, 0.82)** | .007 |  |
| ≥75 | | 1.00 | | 1.24 (0.69, 2.56) | | 0.59 (0.29, 1.21) | 0.45(0.19, 1.08) | .030 |  |
| COPD-related inpatient visits | |  | |  | |  |  |  |  |
| 0 | | 1.00 | | 1.08(0.63, 1.84) | | 0.61 (0.34, 1.09) | 0.47(0.27, 0.82)** | .003 |  |
| 1 | | 1.00 | | 0.48(0.14, 1.67) | | 0.67 (0.25, 1.84) | 0.21(0.03, 1.41) | .067 |  |
| ≥2 | | 1.00 | | 2.03(0.59, 7.05) | | 0.48 (0.06, 3.81) | 0.88(0.21, 3.66) | .687 |  |
| CHA2DS2-VASc score | |  | |  | |  |  |  |  |
| 2–3 | | 1.00 | | 0.53 (0.19, 1.48) | | 0.61 (0.27, 1.36) | 0.57(0.30, 1.08) | .058 |  |
| ≥4 | | 1.00 | | 1.27 (0.76, 2.14) | | 0.65 (0.36, 1.19) | 0.37(0.17, 0.81)* | .005 |  |
| Asthma | |  | |  | |  |  |  |  |
| No | | 1.00 | | 1.04 (0.54, 2.03) | | 0.99 (0.54, 1.80) | 0.55(0.27, 1.12) | .148 |  |
| Yes | | 1.00 | | 1.01 (0.55, 1.85) | | 0.37 (0.17, 0.82)* | 0.38(0.19, 0.76)** | < .001 |  |
| HF | |  | |  | |  |  |  |  |
| No | | 1.00 | | 1.21 (0.75, 1.96) | | 0.67 (0.39, 1.14) | 0.46(0.27, 0.80)** | .003 |  |
| Yes | | 1.00 | | 0.33 (0.07, 1.47) | | 0.50 (0.15, 1.67) | 0.49(0.15, 1.59) | .149 |  |
| AF | |  | |  | |  |  |  |  |
| No | | 1.00 | | 0.94 (0.56, 1.58) | | 0.67 (0.40, 1.12) | 0.43(0.25, 0.74)** | .001 |  |
| Yes | | 1.00 | | 1.33 (0.51, 3.47) | | 0.44 (0.10, 1.97) | 0.70(0.21, 2.37) | .365 |  |
| Ischemic heart disease | |  | |  | |  |  |  |  |
| No | | 1.00 | | 0.86 (0.48, 1.54) | | 0.58 (0.32, 1.05) | 0.41(0.22, 0.75)** | .001 |  |
| Yes | | 1.00 | | 1.43 (0.67, 3.05) | | 0.79 (0.34, 1.85) | 0.65(0.27, 1.54) | .290 |  |
| Angina | |  | |  | |  |  |  |  |
| No | | 1.00 | | 0.94 (0.57, 1.55) | | 0.63 (0.37, 1.06) | 0.41(0.23, 0.72)** | < .001 |  |
| Yes | | 1.00 | | 1.72 (0.58, 5.14) | | 0.75 (0.20, 2.83) | 1.10(0.37, 3.24) | .952 |  |
| Peripheral vascular disease | |  | |  | |  |  |  |  |
| No | | 1.00 | | 1.10 (0.68, 1.79) | | 0.68 (0.41, 1.14) | 0.47(0.28, 0.80)** | .003 |  |
| Yes | | 1.00 | | 0.53 (0.14, 2.01) | | 0.37 (0.09, 1.56) | 0.47(0.10, 2.09) | .148 |  |
| Hypertension | |  | |  | |  |  |  |  |
| No | | 1.00 | | 0.82 (0.34, 1.98) | | 0.39 (0.14, 1.11) | 0.66(0.35, 1.27) | .083 |  |
| Yes | | 1.00 | | 1.08 (0.63, 1.85) | | 0.74 (0.42, 1.29) | 0.35(0.17, 0.73)** | .003 |  |
| Diabetes | |  | |  | |  |  |  |  |
| No | | 1.00 | | 0.84 (0.47, 1.50) | | 0.42 (0.21, 0.81)** | 0.39(0.21, 0.73)** | < .001 |  |
| Yes | | 1.00 | | 1.59 (0.74, 3.41) | | 1.28 (0.60, 2.69) | 0.68(0.28, 1.65) | .652 |  |
| Renal failure | |  | |  | |  |  |  |  |
| No | | 1.00 | | 0.79 (0.46, 1.35) | | 0.61 (0.36, 1.02) | 0.38(0.22, 0.67)*** | < .001 |  |
| Yes | | 1.00 | | 2.99 (0.99, 9.16) | | 1.05 (0.28, 3.93) | 1.48(0.48, 4.55) | .568 |  |
| Chronic liver disease | |  | |  | |  |  |  |  |
| No | | 1.00 | | 0.92(0.54, 1.56) | | 0.61 (0.35, 1.04) | 0.48(0.28, 0.83)** | .003 |  |
| Yes | | 1.00 | | 1.57(0.65, 3.78) | | 0.78 (0.26, 2.29) | 0.43(0.13, 1.40) | .135 |  |
| Influenza infection | |  | |  | |  | |  |  |
| No | | 1.00 | | 0.74(0.54, 1.00)* | | 7.57(0.09, 648.00) | | 0.47(0.20, 1.06) | .004 |
| Yes | | 1.00 | | 1.08(0.61, 1.91) | | 0.66(0.35, 1.23) | | 0.55(0.29, 1.03) | .049 |

*: *P* < .05 **: *P* < .01 ***: *P* < .001

COPD, chronic obstructive pulmonary disease; CI, confidence interval; aHR, adjusted hazard ratio; HF, heart failure; AMI, acute myocardial infarction; AF, atrial fibrillation; RAASi, renin–angiotensin–aldosterone system inhibitors

†The main model was adjusted for propensity score, and the use of aspirin, statins, RAASi, and metformin, duration between the COPD diagnosis date and index date.

*: P < .05 **: P < .01 ***: P < .001

‡A subdistribution hazard model was used as a sensitivity analysis to account for death as a competing risk.

**Table 2.** **Sensitivity Analysis of the Adjusted Hazard Ratios of Vaccination in Ischemic Stroke Risk Reduction**

|  | | Unvaccinated | | Vaccinated | | | | *P* for Trend |  |
| --- | --- | --- | --- | --- | --- | --- | --- | --- | --- |
|  |  |  |  | 1 | | 2–3 | **≥**4 |  |  |
|  |  | aHR  (95% CI) | | aHR  (95% CI) | | aHR  (95% CI) | aHR  (95% CI) |  |  |
| **Unadjusted** | | 1.00 | | 1.01 (0.83, 1.24) | | 0.75 (0.62, 0.88)*** | 0.26(0.21, 0.33)*** | < .001 |  |
| Main model † | | 1.00 | | 1.03 (0.85, 1.26) | | 0.75 (0.63, 0.90)** | 0.28(0.22, 0.36)*** | < .001 |  |
| Competing Risk model‡ | | 1.00 | | 0.89 (0.73, 1.09) | | 0.81 (0.68, 0.96)* | 0.36(0.29, 0.45)*** | < .001 |  |
| **Subgroup effects** | |  | |  | |  |  |  |  |
| Age, years | |  | |  | |  |  |  |  |
| 65–74 | | 1.00 | | 1.06 (0.78, 1.43) | | 0.96 (0.75, 1.22) | 0.36(0.27, 0.49)*** | < .001 |  |
| ≥75 | | 1.00 | | 0.77 (0.58, 1.02) | | 0.68 (0.52, 0.88)** | 0.36(0.25, 0.52)*** | < .001 |  |
| COPD-related inpatient visits | |  | |  | |  |  |  |  |
| 0 | | 1.00 | | 0.83(0.65, 1.07) | | 0.82 (0.68, 1.01) | 0.33(0.26, 0.43)*** | < .001 |  |
| 1 | | 1.00 | | 1.13(0.72, 1.79) | | 0.59 (0.36, 0.98)* | 0.32(0.15, 0.67)** | < .001 |  |
| ≥2 | | 1.00 | | 0.78(0.45, 1.38) | | 0.83 (0.49, 1.40) | 0.66(0.36, 1.21) | .157 |  |
| CHA2DS2-VASc score | |  | |  | |  |  |  |  |
| 2–3 | | 1.00 | | 0.88 (0.58, 1.34) | | 0.94 (0.68, 1.28) | 0.37(0.25, 0.53)*** | < .001 |  |
| ≥4 | | 1.00 | | 0.84 (0.66, 1.06) | | 0.74 (0.60, 0.91)** | 0.38(0.28, 0.50)*** | < .001 |  |
| Asthma | |  | |  | |  |  |  |  |
| No | | 1.00 | | 0.78 (0.58, 1.06) | | 0.76 (0.59, 0.98)* | 0.28(0.20, 0.40)*** | < .001 |  |
| Yes | | 1.00 | | 0.98 (0.74, 1.30) | | 0.85 (0.66, 1.08) | 0.43(0.32, 0.57)*** | < .001 |  |
| HF | |  | |  | |  |  |  |  |
| No | | 1.00 | | 0.82 (0.65, 1.03) | | 0.82 (0.68, 0.99)* | 0.36(0.28, 0.45)*** | < .001 |  |
| Yes | | 1.00 | | 1.24 (0.81, 1.91) | | 0.74 (0.46, 1.20) | 0.34(0.17, 0.67)** | .001 |  |
| AF | |  | |  | |  |  |  |  |
| No | | 1.00 | | 1.01 (0.80, 1.26) | | 0.86 (0.71, 1.04) | 0.34(0.26, 0.44)*** | < .001 |  |
| Yes | | 1.00 | | 0.53 (0.32, 0.87)* | | 0.69 (0.46, 1.03) | 0.50(0.33, 0.78)** | < .001 |  |
| Ischemic heart disease | |  | |  | |  |  |  |  |
| No | | 1.00 | | 0.87 (0.66, 1.15) | | 0.87 (0.69, 1.09) | 0.38(0.29, 0.49)*** | < .001 |  |
| Yes | | 1.00 | | 0.87 (0.64, 1.18) | | 0.70 (0.53, 0.92)* | 0.33(0.22, 0.49)*** | < .001 |  |
| Angina | |  | |  | |  |  |  |  |
| No | | 1.00 | | 0.82 (0.66, 1.03) | | 0.81 (0.67, 0.97)* | 0.35(0.27, 0.44)*** | < .001 |  |
| Yes | | 1.00 | | 1.55 (0.92, 2.63) | | 0.80 (0.46, 1.39) | 0.49(0.24, 1.00) | .048 |  |
| Peripheral vascular disease | |  | |  | |  |  |  |  |
| No | | 1.00 | | 0.91 (0.73, 1.13) | | 0.79 (0.66, 0.95)* | 0.34(0.27, 0.43)*** | < .001 |  |
| Yes | | 1.00 | | 0.82 (0.42, 1.58) | | 0.97 (0.55, 1.71) | 0.64(0.32, 1.30) | .312 |  |
| Hypertension | |  | |  | |  |  |  |  |
| No | | 1.00 | | 0.97 (0.64, 1.46) | | 0.94 (0.68, 1.31) | 0.43(0.29, 0.62)*** | < .001 |  |
| Yes | | 1.00 | | 0.82 (0.65, 1.04) | | 0.74 (0.60, 0.91)** | 0.34(0.26, 0.45)*** | < .001 |  |
| Diabetes | |  | |  | |  |  |  |  |
| No | | 1.00 | | 0.81 (0.63, 1.05) | | 0.76 (0.62, 0.94)* | 0.35(0.27, 0.46)*** | < .001 |  |
| Yes | | 1.00 | | 1.04 (0.74, 1.45) | | 0.91 (0.66, 1.25) | 0.39(0.26, 0.58)*** | < .001 |  |
| Renal failure | |  | |  | |  |  |  |  |
| No | | 1.00 | | 0.91 (0.73, 1.14) | | 0.79 (0.65, 0.96)* | 0.37(0.29, 0.46)*** | < .001 |  |
| Yes | | 1.00 | | 0.81 (0.49, 1.33) | | 0.85 (0.55, 1.30) | 0.31(0.16, 0.59)*** | < .001 |  |
| Chronic liver disease | |  | |  | |  |  |  |  |
| No | | 1.00 | | 0.86 (0.68, 1.09) | | 0.77 (0.63, 0.94)** | 0.37(0.29, 0.48)*** | < .001 |  |
| Yes | | 1.00 | | 0.99 (0.64, 1.52) | | 0.97 (0.66, 1.43) | 0.29(0.17, 0.50)*** | < .001 |  |
| Influenza infection | |  | |  | |  | |  |  |
| No | | 1.00 | | 0.83(0.66, 1.05) | | 0.84(0.68, 1.02) | | 0.39(0.30, 0.50)*** | < .001 |
| Yes | | 1.00 | | 1.17(0.76, 1.80) | | 0.66(0.46, 0.96)* | | 0.25(0.16, 0.40)*** | < .001 |

*: *P* < .05 **: *P* < .01 ***: *P* < .001

COPD, chronic obstructive pulmonary disease; CI, confidence interval; aHR, adjusted hazard ratio; HF, heart failure; AMI, acute myocardial infarction; AF, atrial fibrillation; RAASi, renin–angiotensin–aldosterone system inhibitors

†The main model was adjusted for propensity score, and the use of aspirin, statins, RAASi, and metformin, duration between the COPD diagnosis date and index date.

*: P < .05 **: P < .01 ***: P < .001

‡A subdistribution hazard model was used as a sensitivity analysis to account for death as a competing risk.

**Table 3.** **Sensitivity Analysis of the Adjusted Hazard Ratios of Vaccination in Undefined Stroke Risk Reduction**

|  | | Unvaccinated | | Vaccinated | | | | *P* for Trend |  |
| --- | --- | --- | --- | --- | --- | --- | --- | --- | --- |
|  |  |  |  | 1 | | 2–3 | **≥**4 |  |  |
|  |  | aHR  (95% CI) | | aHR  (95% CI) | | aHR  (95% CI) | aHR  (95% CI) |  |  |
| **Unadjusted** | | 1.00 | | 1.14 (0.82, 1.59) | | 0.58 (0.41, 0.82)** | 0.38(0.26, 0.54)*** | < .001 |  |
| Main model † | | 1.00 | | 1.17 (0.84, 1.63) | | 0.60 (0.42, 0.85)** | 0.41(0.29, 0.60)*** | < .001 |  |
| Competing Risk model‡ | | 1.00 | | 1.04 (0.75, 1.45) | | 0.65 (0.46, 0.92)* | 0.54(0.39, 0.76)*** | < .001 |  |
| **Subgroup effects** | |  | |  | |  |  |  |  |
| Age, years | |  | |  | |  |  |  |  |
| 65–74 | | 1.00 | | 1.06 (0.65, 1.72) | | 0.55 (0.34, 0.92)* | 0.42(0.27, 0.67)*** | < .001 |  |
| ≥75 | | 1.00 | | 1.05 (0.66, 1.66) | | 0.77 (0.47, 1.25) | 0.75(0.46, 1.24) | .164 |  |
| COPD-related inpatient visits | |  | |  | |  |  |  |  |
| 0 | | 1.00 | | 0.92(0.60, 1.42) | | 0.74 (0.50, 1.11) | 0.53(0.36, 0.78)** | < .001 |  |
| 1 | | 1.00 | | 0.74(0.20, 1.71) | | 0.33 (0.08, 1.43) | 0.88(0.33, 2.38) | .429 |  |
| ≥2 | | 1.00 | | 1.48(0.81, 2.68) | | 0.54 (0.23, 1.26) | 0.43(0.16, 1.17) | .044 |  |
| CHA2DS2-VASc score | |  | |  | |  |  |  |  |
| 2–3 | | 1.00 | | 0.72 (0.33, 1.60) | | 0.73 (0.39, 1.36) | 0.44(0.25, 0.78)** | .005 |  |
| ≥4 | | 1.00 | | 1.09 (0.75, 1.58) | | 0.61 (0.40, 0.93)* | 0.64(0.42, 0.97)* | .006 |  |
| Asthma | |  | |  | |  |  |  |  |
| No | | 1.00 | | 0.85 (0.53, 1.37) | | 0.63 (0.39, 1.02) | 0.43(0.26, 0.71)** | < .001 |  |
| Yes | | 1.00 | | 1.31 (0.82, 2.10) | | 0.68 (0.40, 1.14) | 0.67(0.42, 1.06) | .041 |  |
| HF | |  | |  | |  |  |  |  |
| No | | 1.00 | | 1.09 (0.75, 1.58) | | 0.69 (0.47, 1.01) | 0.59(0.41, 0.84)** | .001 |  |
| Yes | | 1.00 | | 0.82 (0.39, 1.71) | | 0.52 (0.22, 1.23) | 0.30(0.10, 0.95)* | .012 |  |
| AF | |  | |  | |  |  |  |  |
| No | | 1.00 | | 0.90 (0.61, 1.32) | | 0.61 (0.41, 0.91)* | 0.54(0.38, 0.78)** | < .001 |  |
| Yes | | 1.00 | | 1.70 (0.85, 1.38) | | 0.85 (0.38, 1.87) | 0.61(0.26, 1.42) | .252 |  |
| Ischemic heart disease | |  | |  | |  |  |  |  |
| No | | 1.00 | | 1.13 (0.74, 1.13) | | 0.61 (0.38, 0.96)* | 0.47(0.31, 0.73)*** | < .001 |  |
| Yes | | 1.00 | | 0.92 (0.53, 1.59) | | 0.70 (0.41, 1.20) | 0.67(0.38, 1.16) | .081 |  |
| Angina | |  | |  | |  |  |  |  |
| No | | 1.00 | | 1.05 (0.73, 1.49) | | 0.70 (0.38, 1.00) | 0.55(0.39, 0.78)*** | < .001 |  |
| Yes | | 1.00 | | 1.18 (0.49, 2.87) | | 0.39 (0.12, 1.35) | 0.48(0.15, 1.54) | .084 |  |
| Peripheral vascular disease | |  | |  | |  |  |  |  |
| No | | 1.00 | | 1.01 (0.71, 1.44) | | 0.63 (0.43, 0.90)* | 0.51(0.36, 0.72)*** | < .001 |  |
| Yes | | 1.00 | | 1.20 (0.43, 3.37) | | 1.08 (0.35, 3.32) | 1.40(0.44, 4.44) | .614 |  |
| Hypertension | |  | |  | |  |  |  |  |
| No | | 1.00 | | 0.64 (0.30, 1.35) | | 0.62 (0.33, 1.17) | 0.42(0.33, 0.79)** | .003 |  |
| Yes | | 1.00 | | 1.19 (0.82, 1.74) | | 0.66 (0.44, 1.01) | 0.63(0.42, 0.94)* | .007 |  |
| Diabetes | |  | |  | |  |  |  |  |
| No | | 1.00 | | 1.00 (0.65, 1.54) | | 0.78 (0.52, 1.16) | 0.64(0.43, 0.94)* | .015 |  |
| Yes | | 1.00 | | 1.08 (0.64, 1.82) | | 0.39 (0.19, 0.82)* | 0.37(0.18, 0.76)** | < .001 |  |
| Renal failure | |  | |  | |  |  |  |  |
| No | | 1.00 | | 1.02 (0.71, 1.47) | | 0.65 (0.45, 0.94)* | 0.52(0.37, 0.74)*** | < .001 |  |
| Yes | | 1.00 | | 1.12 (0.47, 2.65) | | 0.59 (0.21, 1.69) | 0.66(0.21, 2.02) | .288 |  |
| Chronic liver disease | |  | |  | |  |  |  |  |
| No | | 1.00 | | 0.96 (0.65, 1.44) | | 0.67 (0.45, 0.98)* | 0.56(0.38, 0.82)** | < .001 |  |
| Yes | | 1.00 | | 1.24 (0.66, 2.31) | | 0.56 (0.25, 1.24) | 0.42(0.20, 0.90)* | .010 |  |
| Influenza infection | |  | |  | |  | |  |  |
| No | | 1.00 | | 1.01(0.69, 1.47) | | 0.50(0.32, 0.78)** | | 0.59(0.40, 0.87)** | < .001 |
| Yes | | 1.00 | | 1.07(0.52, 2.20) | | 0.95(0.53, 1.71) | | 0.36(0.18, 0.73)** | .007 |

*: *P* < .05 **: *P* < .01 ***: *P* < .001

COPD, chronic obstructive pulmonary disease; CI, confidence interval; aHR, adjusted hazard ratio; HF, heart failure; AMI, acute myocardial infarction; AF, atrial fibrillation; RAASi, renin–angiotensin–aldosterone system inhibitors

†The main model was adjusted for propensity score, and the use of aspirin, statins, RAASi, and metformin, duration between the COPD diagnosis date and index date.

*: P < .05 **: P < .01 ***: P < .001

‡A subdistribution hazard model was used as a sensitivity analysis to account for death as a competing risk.

Table 4 Analysis of non-interrupted and interrupted vaccination

|  | Unvaccinated | Vaccinated | | |
| --- | --- | --- | --- | --- |
|  |  | **1 time** | **Interruption^a^** | **Noninterruption^b^** |
|  | Adjusted HR  (95%C.I.) | Adjusted HR  (95%C.I.) | Adjusted HR  (95%C.I.) | Adjusted HR  (95%C.I.) |
| Stroke |  |  |  |  |
| Unadjusted | 1.00 | 1.05(0.90, 1.23) | 0.41(0.35, 0.47)*** | 0.57(0.47, 0.70)*** |
| Main model † | 1.00 | 1.07(0.92, 1.26) | 0.44(0.38, 0.51)*** | 0.60(0.49, 0.73)*** |
| Hemorrhagic stroke |  |  |  |  |
| Unadjusted | 1.00 | 1.15(0.73, 1.81) | 0.34(0.21, 0.53)*** | 0.62(0.35, 1.08) |
| Main model † | 1.00 | 1.18(0.75, 1.85) | 0.37(0.23, 0.59)*** | 0.67(0.38, 1.18) |
| Ischemic stroke |  |  |  |  |
| Unadjusted | 1.00 | 1.01(0.83, 1.23) | 0.44(0.37, 0.52)*** | 0.50(0.39, 0.65)*** |
| Main model † | 1.00 | 1.03(0.84, 1.25) | 0.47(0.39, 0.56)*** | 0.52(0.40, 0.67)*** |
| Undefined stroke |  |  |  |  |
| Unadjusted | 1.00 | 1.14(0.82, 1.58) | 0.36(0.26, 0.50)*** | 0.76(0.52, 1.12) |
| Main model † | 1.00 | 1.17(0.82, 1.62) | 0.39(0.28, 0.54)*** | 0.79(0.54, 1.17) |

* : *P* < .05 ** : *P* < .01 *** : *P* < .001

aHR: adjusted hazard ratio

^a^Interruption: defined as receiving more than two vaccinations with any interruption during the follow-up period.

^b^Noninterruption: defined as receiving more than two yearly vaccinations without any interruption during the follow-up period.

† The main model was adjusted for propensity score, and the use of aspirin, statins, RAASi, and metformin, duration between the COPD diagnosis date and index date.

Table 5 Analysis of non-interrupted vaccination

| **Noninterrupted vaccination^a^** | Unvaccinated | **Vaccination times** | | |
| --- | --- | --- | --- | --- |
|  |  | **2** | **3** | **≥ 4** |
|  | Adjusted HR  (95%C.I.) | Adjusted HR  (95%C.I.) | Adjusted HR  (95%C.I.) | Adjusted HR  (95%C.I.) |
| Stroke |  |  |  |  |
| Unadjusted | 1.00 | 0.78(0.55, 1.11) | 0.82(0.54, 1.24) | 0.49(0.35, 0.68)*** |
| Main model † | 1.00 | 0.79(0.55, 1.12) | 0.84(0.55, 1.28) | 0.51(0.36, 0.72)*** |
| Hemorrhagic stroke |  |  |  |  |
| Unadjusted | 1.00 | 0.86(0.31, 2.35) | 0.57(0.13, 2.47) | 0.72(0.30, 1.75) |
| Main model † | 1.00 | 0.92(0.34, 2.52) | 0.64(0.15, 2.79) | 0.81(0.33, 1.99) |
| Ischemic stroke |  |  |  |  |
| Unadjusted | 1.00 | 0.77(0.50, 1.18) | 0.73(0.43, 1.24) | 0.36(0.23, 0.57)*** |
| Main model † | 1.00 | 0.77(0.50, 1.19) | 0.78(0.46, 1.32) | 0.39(0.24, 0.62)*** |
| Undefined stroke |  |  |  |  |
| Unadjusted | 1.00 | 0.78(0.36, 1.71) | 1.23(0.56, 2.69) | 0.77(0.42, 1.44) |
| Main model † | 1.00 | 0.75(0.34, 1.64) | 1.12(0.51, 2.47) | 0.75(0.40, 1.40) |

* : *P* < .05 ** : *P* < .01 *** : *P* < .001

aHR: adjusted hazard ratio

† The main model was adjusted for propensity score, and the use of aspirin, statins, RAASi, and metformin, duration between the COPD diagnosis date and index date.

Table 6 Analysis of interrupted vaccination

| **Interrupted vaccination^a^** | Unvaccinated | **Vaccination times** | | |
| --- | --- | --- | --- | --- |
|  |  | **2** | **3** | **≥ 4** |
|  | Adjusted HR  (95%C.I.) | Adjusted HR  (95%C.I.) | Adjusted HR  (95%C.I.) | Adjusted HR  (95%C.I.) |
| Stroke |  |  |  |  |
| Unadjusted | 1.00 | 0.74(0.59, 0.93)* | 0.49(0.37, 0.64)*** | 0.22(0.17, 0.27)*** |
| Main model † | 1.00 | 0.78(0.62, 0.98)* | 0.48(0.37, 0.63)*** | 0.23(0.18, 0.29)*** |
| Hemorrhagic stroke |  |  |  |  |
| Unadjusted | 1.00 | 0.76(0.39, 1.50) | 0.21(0.07, 0.67)** | 0.19(0.10, 0.38)*** |
| Main model † | 1.00 | 0.82(0.41, 1.62) | 0.21(0.07, 0.69)** | 0.22(0.11, 0.43)*** |
| Ischemic stroke |  |  |  |  |
| Unadjusted | 1.00 | 0.90(0.69, 1.17) | 0.53(0.38, 0.73)*** | 0.22(0.16, 0.29)*** |
| Main model † | 1.00 | 0.93(0.72, 1.22) | 0.52(0.37, 0.71)*** | 0.22(0.17, 0.30)*** |
| Undefined stroke |  |  |  |  |
| Unadjusted | 1.00 | 0.29(0.14, 0.60)*** | 0.51(0.29, 0.90)* | 0.23(0.14, 0.37)*** |
| Main model † | 1.00 | 0.31(0.15, 0.65)** | 0.52(0.29, 0.90)* | 0.25(0.16, 0.40)*** |

* : *P* < .05 ** : *P* < .01 *** : *P* < .001

aHR: adjusted hazard ratio

† The main model was adjusted for propensity score, and the use of aspirin, statins, RAASi, and metformin, duration between the COPD diagnosis date and index date.
