# Supplementary figures and images for: Influenza Vaccination and Risk of Stroke in Women With Chronic Obstructive Pulmonary Disease: A Nationwide, Population-Based, Propensity-Matched Cohort Study
Source: Front Med (Lausanne). 2022 May 19;9:811021. doi: 10.3389/fmed.2022.811021 (PMC9160371; doi:10.3389/fmed.2022.811021)

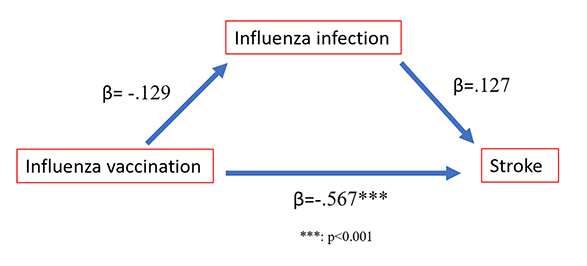

Supplement: Supplementary file 2 [file Image_1.TIF]
